# Evaluation of Cytotoxic Activity of Cell Biomass from *Eryngium planum* and *Lychnis flos-cuculi* on Melanoma Cancer Cell

**DOI:** 10.3390/molecules29215158

**Published:** 2024-10-31

**Authors:** Anastasia Aliesa Hermosaningtyas, Ewa Totoń, Natalia Lisiak, Dariusz Kruszka, Anna Budzianowska, Małgorzata Kikowska

**Affiliations:** 1 Laboratory of Pharmaceutical Biology and Biotechnology, Department and Division of Practical Cosmetology and Skin Diseases Prophylaxis, Poznan University of Medical Sciences, Collegium Pharmaceuticum, 3 Rokietnicka St., 60-806 Poznan, Poland; abudzian@ump.edu.pl; 2 Doctoral School, Poznan University of Medical Sciences, 70 Bukowska St., 60-812 Poznan, Poland; 3 Department of Clinical Chemistry and Molecular Diagnostics, Poznan University of Medical Sciences, Collegium Pharmaceuticum, 3 Rokietnicka St., 60-806 Poznan, Poland; etoton@ump.edu.pl (E.T.); nlisiak@ump.edu.pl (N.L.); 4 Institute of Plant Genetics, Polish Academy of Sciences, 34 Strzeszyńska St., 60-479 Poznan, Poland; dkru@igr.poznan.pl

**Keywords:** melanoma cell, cytotoxic activity, flat sea holly, ragged robin, callus, plant cell cultures

## Abstract

Melanoma is a malignant neoplasm of melanocytes in the skin, and its occurrence is increasing annually. Plant-based products contain active compounds with low toxicity and are accessible alternatives for melanoma cancer treatment. The biotechnology approach for obtaining plant-based products provides continuity and allows the high-yield production of phytochemically uniform biomass. The callus biomass of *Eryngium planum* L. and *Lychnis flos-cuculi* L. was induced on Murashige and Skoog (MS) medium supplemented with growth regulators. A combination of 3.0 mg/L of 3,6-dichloro-2-methoxybenzoic acid (dicamba) and 0.3 mg/L of 1-phenyl-3-(1,2,3-thiadiazol-5-yl)urea—(thidiazuron) was used to obtain *E. planum* callus. Meanwhile, the callus of *L. flos-cuculi* was cultivated on MS medium with 2.0 mg/L of 2,4-dichlorophenoxyacetic acid (2,4-D). Methanolic extracts (EpME and LFcME), including 40% MeOH fractions (Ep40MF and LFc40MF) and 80% MeOH fractions (Ep80MF and LFc80MF), of *E. planum* and *L. flos-cuculi* cell biomass were prepared. Their cytotoxicity activity was assessed in human fibroblast cells (MRC-5) and human melanoma cells (MeWo) by direct cell counting and 3-[4,5-dimethylthiazol-2-yl]-2,5 diphenyl tetrazolium bromide (MTT) assay. Qualitative analyses using thin-layer chromatography and UPLC-HRMS/MS chromatograms showed the presence of phenolic acids and saponins within the extracts and fractions of both cell biomasses. LFc80MF and Ep80MF showed the strongest toxicity against the MeWo cell line, with IC_50_ values of 47 ± 0.5 and 52 ± 4 μg/mL after 72 h of treatment. EpME and LFcME had IC_50_ values of 103 ± 4 and 147 ± 4 µg/mL, respectively. On the other hand, Ep40MF and LFc40MF were less toxic against the MeWo cell line compared to the extracts and 80% MeOH fractions, with IC_50_ values of 145 ± 10 and 172 ± 7 µg/mL. This study suggests that the obtained extracts and fractions of *E. planum* and *L. flos-cuculi* cell biomass potentially possess significant cytotoxic activity against MeWo cells, which work in a time and dose-dependent manner. Although the extracts and 80% MeOH fractions were more potent, the 40% MeOH was shown to be more selective against the MeWo than the control MRC-5 cells.

## 1. Introduction

Melanoma is a malignant neoplasm of melanocytes commonly found in the skin and has the worst prognosis among skin cancers [1]. The incidence of melanoma cancer is increasing annually [2]. Melanoma of the skin climbed up from the 19th position in 2020 to the 17th position in 2022 among the most common cancer cases worldwide, as reported by the GLOBOCAN [3,4]. In the United States (1990–2019), the incidence and prevalence of melanoma increased by 4.4 (from 12.6 to 17.0) and 38.5 (from 99.5 to 138) per 100,000 population, respectively. Fortunately, the mortality rates have gradually decreased over the past 15 years [5]. Similarly, the incidence rate of melanoma is about <25 cases per 100,000 individuals in Europe [6]. Further, a study conducted in Poland between 2000 and 2020 calculated that the mortality due to skin melanoma increased from 3.60 to 4.03 per 100,000 people [1]. The available melanoma treatments range from conventional excisional surgery and chemotherapy to targeted systemic immunotherapy treatments or the combination of treatments (BRAF inhibitor, MEK inhibitor, anti-PD-1 monoclonal antibody, and anti-CTLA-4 monoclonal antibody) [7,8]. However, chemoresistance and adverse side effects that could cause the cancer to relapse are significant problems associated with these treatments [9]. Therefore, alternatives for melanoma cancer treatment without profound side effects and which are accessible to patients are needed.

Plant-based products (extracts or compounds) have been proven to be valuable resources against melanoma diseases, to be less toxic for the human body, and to improve patients’ quality of life [9]. The *n*-hexane extract of *Eryngium caucasicum* Trauatv. and its fraction have potential against murine melanoma cell lines (B16F10) through apoptosis induction and the ethanol extract of *Hammada scoparia* (Pomel) Iljin. could inhibit melanogenesis in murine melanoma cell lines (B16) [10,11]. Furthermore, scientific reports showed that single natural compounds, such as curcumin and betulinic acid, worked against human melanoma cell lines (A375 and C8161) via autophagy and caused apoptosis and the reduced cell proliferation of murine melanoma cell line (B164A5), respectively [12,13].

Flat sea holly (*Eryngium planum* L.) and ragged robin (*Lychnis flos-cuculi* L.) are plant species that are native to Poland and contain a wide range of bioactive compounds. *E. planum* has been reported to contain a complex of triterpenoid saponins, phenolic acids, flavonoids, coumarins, acetylenes, and essential oil [14,15,16]. Phytoecdysteroids, triterpene saponins complex, phenolic acids, and flavonoids were identified in *L. flos-cuculi* [17,18,19]. Nevertheless, the instability of cultivation, the time-consuming nature of the process, the potential for a low metabolite content in the raw material, and the intricate extraction process are all considered obstacles to the availability of fresh materials. Thus, plant-cell culture using biotechnology is an exciting alternative to providing material continuity and yield production, as well as phytochemically uniform biomass, for these species [20]. The protocols for the micropropagation of *E. planum* and *L. flos-cuculi* have been developed previously [21,22]. Furthermore, several studies have already performed phytochemical analyses on in vitro-derived organ and cell cultures of these species [21,22,23,24,25,26,27,28]. These secondary metabolites in plant biomass are reported to be associated with pharmacological activities [15,16,18,19,24,25,26,29].

The present study aims to investigate the cytotoxic activity of methanolic extracts and fractions from *E. planum* and *L. flos-cuculi* callus cultures in human melanoma cell lines (MeWo) and human fibroblast cells (MRC-5) using an in vitro MTT assay.

## 2. Results

**Figure 1:**
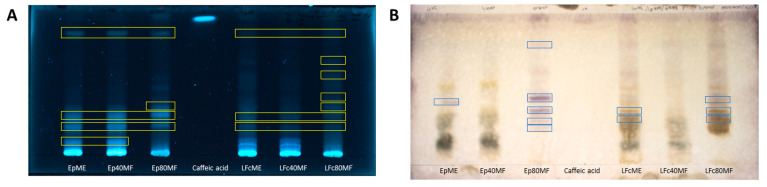
Thin-layer chromatography analysis of *E. planum* and *L. flos-cuculi* cell extracts and fractions on silica plates. The mobile phase used was a mixture of CH_3_COOC_2_H_5_: CH_3_COOH: HCOOH: H_2_O (80:5:5:10 *v*/*v*), and the plate was visualised under 366 nm light (**A**). Additionally, the top phase of C_4_H_9_OH: CH_3_COOH: H_2_O (4:1:5) was used as the mobile phase, and visualisation was completed with white light for saponin detection (**B**). Legend: methanolic *E. planum* (EpME) and *L. flos-cuculi* (LFcME), 40% methanolic fraction (Ep40MF and LFc40MF), 80% methanolic fraction (Ep80MF and LFc80MF).

The initial screening of phytochemical constituents was conducted using the thin-layer chromatography (TLC) technique, as shown in Figure 1 and Table 1. The cell cultures of *E. planum* and *L. flos-cuculi* could produce phenolic acid, exhibited as blue bands in the TLC qualitative analysis (Figure 1A; highlighted in yellow boxes). Intensive bands were visible at Rf values of 0.20, 0.25, and 0.83 for the extract and fractions of *E. planum.* An additional band at an Rf of 0.33 was visible for Ep80MF (Table 1). Similar bands also appeared in LFcME, LFc40 MF, and LFc80MF, with notable bands from LFc80MF being observed at Rf values of 0.33, 0.40, 0.48, 0.53, and 0.67.

Qualitative TLC analyses verified that the cell biomasses could synthesise saponin, which appeared as violet spots on the TLC chromatogram plates after reaction with 1% vanillin and 5% sulphuric acid (Figure 1B; highlighted in blue boxes). Highly intensive bands were observed at an Rf of 0.42 for EpME. Meanwhile, Ep80MF was noticeably richer in saponins, as seen by the emergence of numerous bands at Rf values of 0.22, 0.29, 0.37, 0.44, and 0.78 (Table 1). Saponin compounds were also detected in the *L. flos-cuculi* cell biomass, with bands emerging at Rf values of 0.32 and 0.37 from LFcME and at Rf values of 0.32, 0.37, and 0.44 from LFc80MF.

**Figure 2:**
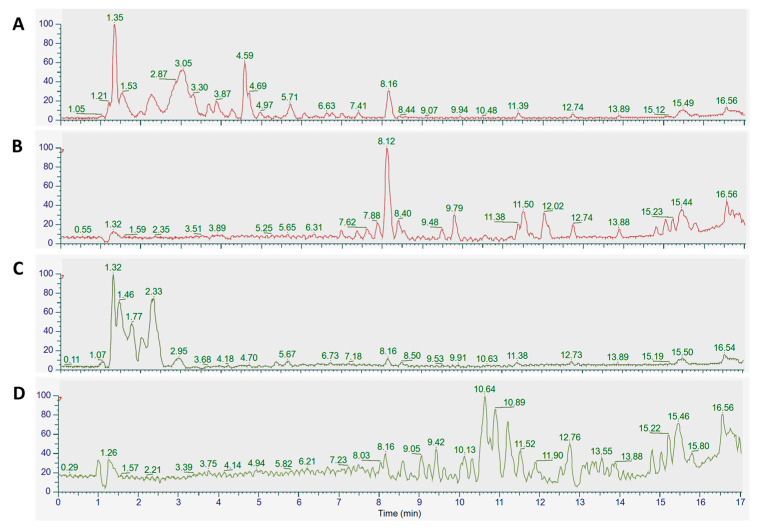
Qualitative analysis of methanolic fractions of Ep40MF (**A**), Ep80MF (**B**), LFc40MF (**C**), and LFc80MF (**D**). The base peak chromatograms in negative ion mode for *E. planum* (red) and *L. flos-cuculi* (green) were obtained using UPLC-HRMS/MS. Legend: methanolic *E. planum* (EpME) and *L. flos-cuculi* (LFcME), 40% methanolic fraction (Ep40MF and LFc40MF), 80% methanolic fraction (Ep80MF and LFc80MF).

Untargeted metabolomic analysis using LC-MS/MS in the negative ion mode was applied (Figure 2). We manually identified phenolic compounds in the 40% methanolic fractions and mostly observed triterpenoid saponins in the 80% methanolic fractions of *E. planum* (Figure 2A,B) and *L. flos-cuculi* (Figure 2C,D), as listed in Table 2.

The methanolic extracts and fractions of *E. planum* and *L. flos-cuculi* cell biomass were investigated against the human melanoma (MeWo) cell line and non-cancerous human fibroblast (MRC-5) cell line to determine their cytotoxicity activity. The cell viability was monitored by performing direct cell counting using the investigated extracts and fractions at concentrations of 10, 50, and 100 μg/mL after 24 h of treatment.

**Figure 3:**
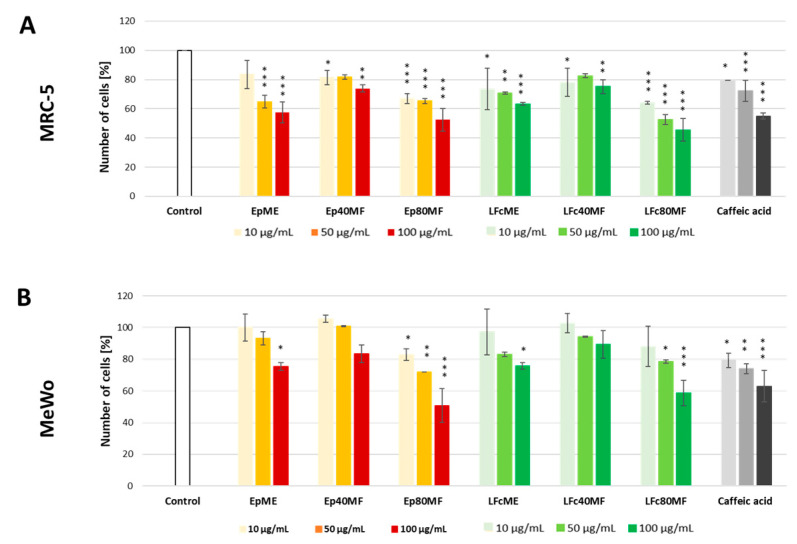
MRC-5 (**A**) and MeWo cells (**B**) were treated with three different concentrations of tested samples for 24 h. Extracts and fractions of *E. planum* and *L. flos-cuculi* are shown in red and green, respectively. The number of cells treated with the reference compound, caffeic acid, is displayed in grey. Untreated cells (white bar) were considered as control samples. The number of cells was counted using the Fuchs-Rosenthal chamber and the phase-contrast microscope Axiovert 40 CFL. Data represent mean ± SD (*n* = 3). * *p* < 0.01 vs. control group, ** *p* < 0.001 vs. control group, *** *p* < 0.0005 vs. control group. Legend: methanolic *E. planum* (EpME) and *L. flos-cuculi* (LFcME), 40% methanolic fraction (Ep40MF and LFc40MF), 80% methanolic fraction (Ep80MF and LFc80MF).

As illustrated in Figure 3, the methanolic extracts from the cell biomass of both plants reduced the MRC-5 and MeWo cell viability in a concentration-dependent manner. A high concentration (100 μg/mL) of EpME significantly reduced the number of MRC-5 and MeWo cells by 43% and 25%, respectively, after 24 h of exposure. Following the treatment of MRC-5 with 10 and 50 μg/mL of EpME, the cell counts were also significantly decreased (17% and 35%, respectively). On the other hand, EpME hindered the MeWo cell number at 50 μg/mL (7% reduction), and no inhibition was observed at 10 μg/mL. However, the changes were insignificant. The cytotoxic impacts on 10 μg/mL MRC-5 and 10 μg/mL MeWo cells were determined by the percentage of cells that degenerated (26–37% and 3–24%, respectively) following a 24-h treatment with LFcME (10–100 μg/mL). A significant decrease in the MRC-5 and MeWo cell numbers was observed when 100 μg/mL of LFcME was added to the cultures (*p*-values < 0.05).

Depending on the concentration, the 40% MeOH fraction of *E. planum* callus (Ep40MF) moderately reduced the number of MRC-5 cells by 18–26%. Interestingly, after the treatment with 10 and 50 μg/mL Ep40MF, the number of MeWo cells slightly increased, although these changes were insignificant compared to the control. The cells were hindered insignificantly only after treatment with 100 μg/mL Ep40MF, which resulted in a 17% decrease. Similarly, 10 μg/mL of Lf40MF slightly increased the number of cells by 3% compared to control. Exposure to 50 and 100 μg/mL of Lf40MF for 24 h minimally decreased the number of both cell lines. There were no significant differences after LFc40MF treatment compared to control.

Changes in viable MRC-5 and MeWo cell numbers were also observed following exposure to the 80% MeOH fractions of *E. planum* (Ep80MF) and *L. flos-cuculi* (LFc80MF). The MRC-5 and MeWo cell counts were narrowed down following treatment with Ep80MF by 33–48% and 17–49%, respectively. LFc80MF decreased the number of cells by 36–55% for the MRC-5 and 12–41% for the MeWo cell lines. Significant changes in the number of cells were visible after exposure to 50–100 μg/mL of Ep80MF and LFc80MF for 24 h. The results showed that 100 μg/mL of Ep80MF or LFc80MF exhibited high cytotoxicity against the human melanoma MeWo cell line (49% and 41%, respectively), comparable to the reference compound, which hindered the number of cells by 37%.

MTT assays were performed to assess further the cytotoxicity effects of the studied extracts and fractions against the human fibroblast MRC-5 and human melanoma MeWo cell lines. Both cell lines were treated with 0–800 μg/mL of the extracts and fractions for 24, 48, and 72 h. As there are no reports yet on activity from the cell biomass of these species against the studied cell lines, a wide range of concentrations was applied. The obtained results are expressed as a percentage of the cell number relative to untreated control (Figure 2A and Figure 3A) and the half-maximal inhibitory concentration (IC_50_) of the studied extracts and fractions are shown as estimated (μg/mL) ± SE in Table 3.

The IC_50_ value is one of the standard methods to determine the cytotoxicity level of a compound, showing the concentration of the tested sample that kills 50% of the cells. In the present study, 80% MeOH fractions of *E. planum* and *L. flos-cuculi* exhibited potent cytotoxic activity in the human melanoma MeWo cell line compared to the extracts and 40% MeOH fractions. The lowest IC_50_ value was demonstrated by LFc80MF to MeWo and decreased over time, reaching 47 ± 0.5 μg/mL after 72 h of treatment. Ep80MF had an IC_50_ value of 52 ± 4 μg/mL in the same treatment period. The 40% MeOH fractions, Ep40MF and LFc40MF, showed poor cytotoxicity in both cell lines. As shown in Table 3, Ep40MF and LFc40MF did not kill MRC-5 or MeWo cells even in high concentrations (IC_50_ > 800 μg/mL), which supports the result from microscopic cell counting. Intriguingly, the obtained data suggested higher selectivity towards the MeWo cells than the MRC-5 cells, which was not shown by either the extracts or 80% MeOH fractions. The lowest IC_50_ value from these fractions was 145 ± 10 μg/mL in the MeWo cell line after exposure to Ep40MF for 72 h. EpME and LFcME both had moderate cytotoxicity impacts on MRC-5 and MeWo at all observation time points.

**Figure 4:**
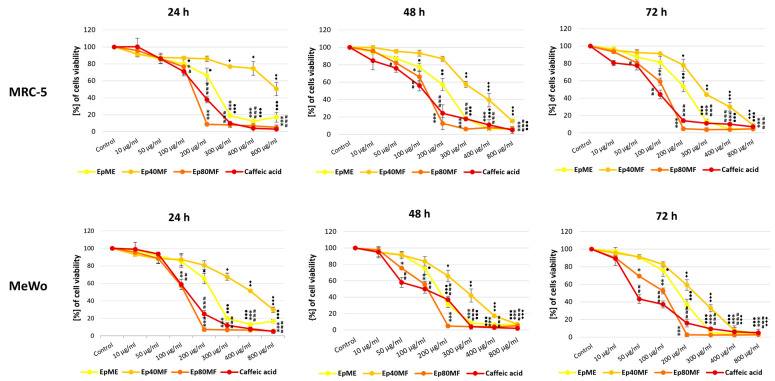
The viability assessment of human fibroblast MRC-5 and human melanoma MeWo cells after treatment with methanolic extracts (EpME), 40% MeOH fraction (Ep40MF), and 80% MeOH fraction (Ep80MF) from *E. planum* callus biomass for 24 h, 48 h, and 72 h presented as line graphs. The cell viability is expressed as a percentage of the non-treated control cells. The mean of three experiments ± SD is shown. Symbols: *, #, •, ♦ for *p* < 0.05; **, ##, ••, ♦♦ for *p* < 0.01; ***, ###, ♦♦♦, ••• for *p* < 0.001. Legend: methanolic *E. planum* (EpME), 40% methanolic fraction (Ep40MF), and 80% methanolic fraction (Ep80MF).

**Figure 5:**
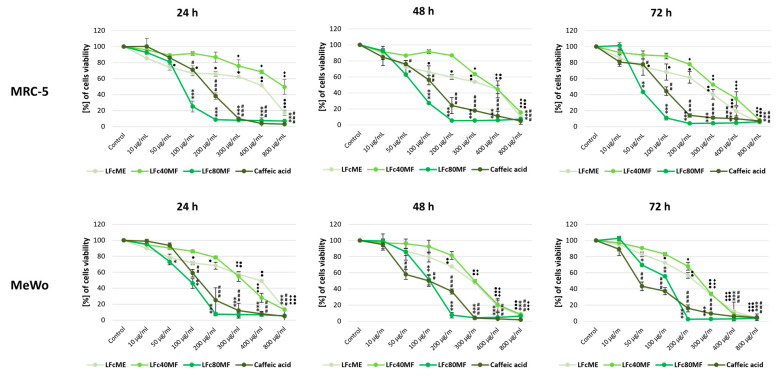
The viability assessment of human fibroblast MRC-5 and human melanoma MeWo cells after treatment with methanolic extracts (LFcME), 40% MeOH fraction (LFc40MF), and 80% MeOH fraction (LFc80MF) from *L. flos-cuculi* callus biomass for 24 h, 48 h, and 72 h presented as line graphs. The cell viability is expressed as a percentage of the non-treated control cells. The mean of three experiments ± SD is shown. Symbols: *, #, •, ♦ for *p* < 0.05; **, ##, ••, ♦♦ for *p* < 0.01; ***, ###, •••, ♦♦♦ for *p* < 0.001. Legend: methanolic *L. flos-cuculi* (LFcME), 40% methanolic fraction (LFc40MF), and 80% methanolic fraction (LFc80MF).

Upon examining the viability assessment via MTT assay, presented in Figure 4 and Figure 5, the results indicated that all extracts and fractions worked in time- and dose-dependent manners.

**Figure 6:**
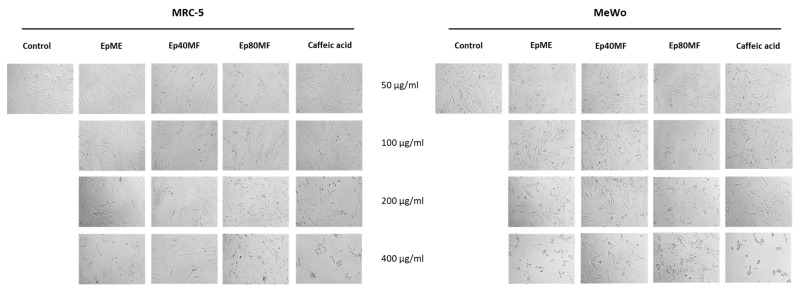
Morphology visualisation showing the effect of studied extracts and fractions with concentration ranging from 50–400 μg/mL on both cell lines after 24 h of treatment. Legend: methanolic *E. planum* (EpME), 40% methanolic fraction (Ep40MF), and 80% methanolic fraction (Ep80MF).

**Figure 7:**
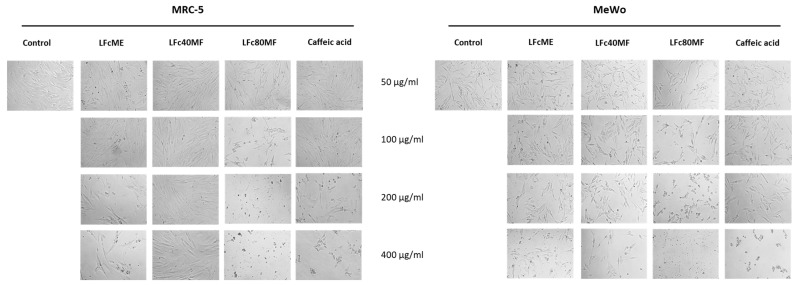
Morphology visualisation showed the effect of studied extracts and fractions with concentration ranging from 50 to 400 μg/mL on both cell lines after 24 h of treatment. Legend: methanolic *L. flos-cuculi* (LFcME), 40% methanolic fraction (LFc40MF), and 80% methanolic fraction (LFc80MF).

Morphological analyses (Figure 6 and Figure 7) presented that EpME, LFcME, Ep80MF, and LFc80MF exhibited significant effects on the morphology of MRC-5 and MeWo cells at concentrations of 200 and 400 μg/mL compared to control untreated cells and the cells treated with Ep40MF and LFc40MF. Following the treatment with extracts and 80% MeOH fractions from both plant calluses, the cells were reduced in number, were shrunken, and had lost their elongated shape. These findings suggested that the methanolic extracts and 80% MeOH fractions were not specific to cancer cells only. On the other hand, the MTT results and morphological analysis implied that the 40% MeOH fractions (Ep40MF and LFc40MF) were effective towards MeWo cells and less sensitive to MRC-5 cells.

## 3. Materials and Methods

### 3.1. Biomass Material and In Vitro Culture Condition

In sterile laminar airflow, *E. planum* plantlets from the culture collection were separated between leaves, roots, and stems. In order to induce the callus, the stem fragments were dissected into smaller pieces and then placed on Murashige and Skoog (MS) [30] solid medium-enriched exogenous supply of 3.0 mg/L 3,6-dichloro-2-methoxybenzoic acid—dicamba (Sigma-Aldrich, St Louis, MO, USA) and 0.3 mg/L 1-phenyl-3-(1,2,3-thiadiazol-5-yl)urea—thidiazuron (Sigma-Aldrich, St Louis, MO, USA), and sucrose 30 g/L. The calluses were maintained and subcultured onto the same hormonal medium variant every four weeks until a stable line of homogeneous calluses was obtained. *L. flos-cuculi* callus was induced from the in vitro-derived stem culture on MS medium supplemented with 2.0 mg/L 2,4-dichlorophenoxyacetic acid (2,4-D) and subcultured until enough materials were available. The collections of callus biomass were kept under controlled conditions—artificial light (16/8 h photoperiod) and temperature (20 ± 1 °C). The obtained biomasses of both species were oven-dried at 30 °C to a constant weight. Callus biomass was assessed morphologically based on colour, structure, and hydration, and microscopically to assess cell viability and type.

### 3.2. Preparation of Extract and Fractions

The dry *E. planum* and *L. flos-cuculi* calluses were extracted three times using 30 mL of 80% MeOH/H_2_O (80:20, *v*/*v*) using an ultrasonic bath for one hour each at 80 °C. The methanolic *E. planum* (EpME) and *L. flos-cuculi* (LFcME) extracts were then evaporated to dryness at the temperature of 40 ± 1 °C and redissolved in distilled water to a 0.2 g/mL concentration. Fractions were prepared using solid-phase extraction Sep-Pak^®^ RP-18 microcolumns (Waters, Milford, MA, USA). Briefly, aqueous solution was applied to the microcolumn in 1 mL increments, then eluted with 5 mL of distilled water combined with 5 mL of freshly prepared 40% MeOH/H_2_O (40:60, *v*/*v*) to yield 40% methanolic fractions. Further, the solution was eluted with 5 mL of 80% MeOH/H_2_O (80:20, *v*/*v*) and pure MeOH combined to obtain the 80% methanolic fractions. The procedure was repeated for each extract to acquire 40% methanolic fractions (Ep40MF and LFc40MF) and 80% methanolic fractions (Ep80MF and LFc80MF), respectively. These fractions underwent evaporation to dryness and were used for cytotoxicity assay.

### 3.3. Phytochemical Evaluation

Preliminary TLC analysis by thin-layer chromatography was done initially to screen the fractions. Five μL aliquots of each extract and fraction were applied to TLC aluminium sheet pre-coated with silica gel 60 (10 × 20 cm^2^, Merck, Darmstadt, Germany) to detect phenolic acids. The plate was then developed in ethyl acetate–acetic acid–formic acid–water (CH_3_COOC_2_H_5_: CH_3_COOH: HCOOH: H_2_O 80:5:5:10 *v*/*v*/*v*/*v*) mixture and later viewed under UV_366_ nm before and after spraying with 0.1% NA (Roth) solution in ethanol. For saponin detection, five μL aliquots of each extract and fraction were applied on TLC aluminium sheets pre-coated with silica gel 60 (10 × 20 cm^2^, Merck, Darmstadt, Germany), developed on mobile phase consisting of 1-butanol–acetic acid–water (C_4_H_9_OH: CH_3_COOH: H_2_O) 40:10:50 *v*/*v*/*v* mixture (top layer), and sprayed with the vanillin–sulfuric acid reagent. Saponins can be seen as a violet-pink colour on the plate.

According to our previous studies, the qualitative analysis of metabolite structures present in extracts and fractions was performed using a high-performance liquid chromatography system coupled to a photodiode array detector/ion-trap mass spectrometer [21,22,23,25,27]. Furthermore, the methanolic extracts of cell biomass from both studied species were analysed using an ultra-performance liquid chromatography system (UPLC, Acquity, Waters) in conjunction with high-resolution tandem mass spectrometry (HRMS/MS, Q Exactive, Thermo, Waltham, MA, USA) [28]. This study examined the methanolic fractions of cell biomass using the same approach. Briefly, samples (5 µL) were separated using an Acquity BEH Shield column (2.1 × 150 mm, 1.7 µm grain size, Waters) at a temperature of 40 °C. The water gradient containing formic acid (A, LC-MS grade, Merck, Darmstadt, Germany) and the acetonitrile gradient (B, LC-MS grade, Merck) were used in the following manner: from 1 to 14 min, a gradient of 5–75% B was applied, followed by a gradient of 75–99% B from 14 to 16 min. From 16 to 19 min, the concentration of B was held constant at 99%. Finally, the concentration of B was returned to 5% over 20 min, with a flow rate of 400 µL/min. The columns were balanced using a 5% B solution for 3 min before the analysis. The PDA detector recorded UV-Vis spectra with a wavelength range of 250–550 nm and a sampling frequency of 20 Hz. The HESI-II, operated in the negative ion mode, had a spray voltage of −3.5 kV and an ion transfer tube temperature of 350 °C. Nitrogen was utilised as a sheath, auxiliary, and sweep gas at flow rates of 35, 10, and 3 arbitrary units, respectively. The auxiliary gas temperature was adjusted to 400 °C, while the S-lens rf level was set at 50. The FullMS scans were acquired within the mass-to-charge ratio (*m*/*z*) range of 100–1000, with a resolution of 70,000 full width at half maximum (FWHM) and a maximum ion-trap period of 200 milliseconds (ms). The ddMS2 (TopN = 5) scans were registered with a resolution of 17,500 FWHM and a maximum ion-trap duration of 100 ms. The higher-energy collisional dissociation collision energy was 35%. The system functioned with Xcalibur 2.2. The metabolites were manually annotated based on exact masses, retention time, and fragmentation spectra.

### 3.4. Cell Line and Culture Methods

The human fetal lung fibroblast cell line MRC-5 (ATCC^®^ CCL-171TM) was cultured in Eagle’s Minimum Essential Medium (EMEM) (Biowest, Nuaillé, France) supplemented with 1% (*v*/*v*) non-essential amino acids (Sigma-Aldrich, Burghausen, Germany) and 10% (*v*/*v*) FBS (Sigma-Aldrich, Burghausen, Germany). The malignant melanoma cell line MeWo (ATCC^®^ HTB-65™) was maintained in a complete growing medium, Eagle’s Minimum Essential Medium (EMEM) (Biowest, Nuaillé, France), supplemented with 10% (*v*/*v*) FBS (Sigma-Aldrich, Burghausen, Germany). Both cell lines were purchased from the American Type Cell Culture Collection (ATCC^®^, Manassas, VA, USA). The cell lines were grown near confluence in 100 × 15 mm cell culture Falcon^®^ Petri dishes (Corning, Warsaw, Poland) in the optimal 90% (*v*/*v*) relative humidity at 37 °C in a humidified chamber with 5% CO_2_ (*v*/*v*) atmosphere. All preparation steps of the culture medium were performed under aseptic conditions. The absence of mycoplasma was checked routinely using the Mycoplasma Stain Kit (Lonza, Monteggio, Switzerland).

### 3.5. Direct Cell Counting

Cell proliferation, comprising an increase in cell number, is a dynamic process balanced by cell division and cell loss. The number of cells was determined manually by counting them manually using a hemocytometer (Fuchs-Rosenthal chamber). In a sterile 6-well plate, the MeWo and the MRC-5 cells were plated at a density of 1 × 10^5^ into each well, in a 2 mL/healthy medium, and incubated overnight under cell culture conditions. Then, both cell lines were treated with EpME, Ep40MF, Ep80MF, LFcME, LFc40MF, LFc80MF, and caffeic acid (reference substance) at concentrations of 10, 50, and 100 μg/mL. The studied extracts and fractions were exposed for 24 h of incubation. Then, cells were passaged and counted using the Fuchs-Rosenthal chamber and the Axiovert 40 CFL phase-contrast microscope (Carl Zeiss, Poznan, Poland). The average cell count of four fields represents the number of cells per mL of cell solution and was used to determine the total number of cells from each well. Three separate experiments were performed, with two repeats for each concentration.

### 3.6. Evaluation of Cytotoxicity Using MTT Assay

The cytotoxicity activity was assessed on both cell lines using MTT (3-(4,5-dimethylthiazol-2-yl)-2,5-diphenyltetrazolium bromide) assay. In a sterile 96-well plate, the cells were plated at a density of 5 × 10^3^ into each well at a concentration of 100 μL/well medium and incubated under cell culture conditions. Both cell lines were treated with EpME, Ep40MF, Ep80MF, LFcME, LFc40MF, LFc80MF, and caffeic acid (as reference for both species) in a 0–800 μg/mL concentration range. All extracts and fractions were dissolved in DMSO, with a final solvent concentration of 0.25% *v*/*v*. Exposure of the studied extracts and fractions to melanoma and fibroblast cell lines was performed for 24, 48, and 72 h of incubation. MTT solution (Sigma-Aldrich, Schnelldorf, Germany) was then added to each well (10 μL; 5 mg/mL) and the 96-well plate was incubated further at 37 °C for four hours, followed by the addition of 100 μL solubilisation buffer (10% SDS in 0.01 M HCl). Microplate Reader Multiscan FC (Thermo Scientific, Waltham, MA, USA) was used to measure the absorbance at 570 nm, with a reference wavelength of 630 nm. Three separate experiments were performed, with three repeats for each concentration. Relative cell viability was determined using the following formula: %viability = (mean of A570–A690 of an experimental group)/(mean of A570–A690 of the control group) × 100%. The viability of the cells was calculated using Excel software (https://www.microsoft.com/en-us/microsoft-365/excel, accessed on 27 June 2024) (Microsoft, Redmond, Washington, DC, USA). The IC_50_ values were obtained using the nonlinear regression program CompuSyn software version 2022 (ComboSyn Inc., Paramus, NJ, USA) (available for free download from www.combosyn.com, accessed on 25 April 2024) and are presented as estimate ± SE [31]. During experiments, the morphology of cell cultures was visualised using an Axiovert 40 CFL inverted microscope (Carl Zeiss, Poznan, Poland).

### 3.7. Statistical Analysis

The obtained data were expressed as mean ± SD from three independent experiments. GraphPad Prism 10.0.3 (GraphPad Software, Boston, MA, USA) was used for statistical analyses. ANOVA and Tukey post-hoc test were used to assess the *p*-value.

## 4. Discussion

The prevalence of melanoma skin cancer has been increasing annually. The primary risk factors for melanoma are family and genetic factors, with ultraviolet radiation and lifestyle choices (such as dietary intake, alcohol consumption, and tanning practices) being secondary contributors [2]. There are various melanoma treatments available for patients, from conventional approaches (surgical, curette and electrodesiccation procedure, and chemotherapy) to targeted therapies (BRAF and MEK inhibitors) and immunotherapies (adoptive cell transfer, immune checkpoint inhibitors, and oncolytic virotherapy) [8,32]. However, some treatments are invasive, expensive, or toxic [33]. Additionally, the majority of patients developed drug resistance within a few months of initiating the BRAF inhibitor treatment, while immunotherapy trials showed a lower response rate and reported severe side effects [34]. Consequently, it is essential to discover novel therapeutics or drug candidates that specifically target cancer cells while minimising the impact on normal, healthy cells and reducing side effects for patients. Phytochemicals, as potential preventive and therapeutic agents, present an alternative solution for cancer [10]. Natural compounds have been extensively studied for their anti-melanoma effects, including tumour growth inhibition, apoptosis induction, angiogenesis and metastasis suppression, and cancer stem cell eradication. Moreover, a number of studies have reported the synergistic activities of phytochemicals and standard anti-melanoma agents, as well as the enhanced effectiveness of their synthetic derivatives and novel formulations. Examples of these natural compounds are polyphenols (flavonoids, curcumin, and resveratrol), organosulfur compounds (sulforaphane), terpenoids (artemisinin, oridonin, and ursolic acid), and saponins that have shown promise as anti-melanoma agents [35].

*Eryngium planum*, the plant of interest in this study, has also been observed for its cytotoxic activity against various cancer cell lines. Notably, the cytotoxic effect of *E. planum* fruit extract against seven human leukemic (ML-1, J-45.01, EOL, HL-60, 1301, C–8166, U-266B1) and two normal (WICL, H-9) cell lines was observed by Bogucka-Kocka et al. (2008). The study reported that the 1301 and HL60 cell lines (viability 62% and 55%, respectively) have longer lifespans than the J45 cell line (viability 9%) after treatment with the ethanol extract of *E. planum* fruit. In contrast, normal T-cells (H9 cell line) and B-cells (WICL cell line) displayed limited viability at 20% and 8%, respectively. Furthermore, the ethanol extract showed a strong apoptosis effect on J45 (96%), C8166 (89%), and U266 (85%) [36]. Additionally, the cytotoxic activity of different fractions, including flavonoid, flavonoid-saponin, saponin, and phenolic acid fractions from the rosette leaves and roots of *E. planum* intact plants was evaluated in a human ovarian cancer cell line using LDH and SRB assays. The study was the first to demonstrate that the barrigenol-type triterpenoid saponin fraction from the roots of *E. planum* inhibited SKOV-3 cells. Among all of the tested fractions, pronounced cytotoxic effects after 24 h of the treatment of cancer cells were demonstrated by saponin fractions at concentrations of 25 and 50 µg/mL. Structure–activity relationship analyses indicated that acylation with angeloyl groups at the C21 or C22 position and the attachment of carbohydrates to the C3 position in triterpenoid saponins are critical to their cytotoxic effects [29]. The anti-cancer potential of other *Eryngium* species was also studied. Hasanbeiglu et al. observed that non-terpenoid compounds from the aerial parts extract of *E. billardieri* were responsible for plant’s cytotoxic activity against B16 melanoma cell lines, through an apoptotic mechanism [37]. Additionally, the essential oils from *E. campestre* and *E. amethystinum* showed high toxicity on the A375 melanoma cell line, with IC_50_ values of 1.57 and 2.78 μg/mL, respectively [38].

Unfortunately, there is currently a lack of research on the potential anti-cancer properties of phytochemicals derived from *Lychnis flos-cuculi.* Nevertheless, other species from the Caryophyllaceae family have been tested for their potential as anti-cancer agents. Ganai et al. reported that *L. coronaria* extract is a promising anti-cancer agent based on their MTT assay results [39]. Phenolic compounds isolated from *L. chalcedonica* presented inhibitory activity against melanoma cell line B16 in an animal model study using the female mice C57BL/6 line [40]. It is worth noting that the population of *E. planum* and *L. flos-cuculi* in their native environment is diminishing owing to extensive enhancement and agricultural expansion [26,41].

The present study highlights the potential of utilising biotechnologically derived cell biomass from *E. planum* and *L. flos-cuculi* cells for pharmaceutical purposes. This approach allows for the sustainable production of plant biomass without threatening these species in their natural habitat and is independent of variable environmental conditions. Furthermore, the results indicated that the extracts and fractions from the cell culture of *E. planum* and *L. flos-cuculi* had anti-tumour activity, mainly by exerting an anti-proliferative effect on the target cells. The findings suggest that this cell biomass might be used as an alternative resource for the development of anti-cancer therapeutics.

The methanolic extracts and fractions were evaluated against human melanoma cells (MeWo). A human lung fibroblast cell line (MRC-5) was chosen to assess the effects of the extracts and fractions from both plants on normal cells. The extracts, namely EpME and LFcME, and their 80% MeOH fractions (Ep80MF and LFc80MF) showed high toxicity towards the melanoma cancer cells. Notably, the toxicity of Ep40MF and LFc40MF (40% MeOH fractions) to MeWo cells was highlighted, contrasting their relative nontoxicity to MRC-5 based on the MTT assay.

Kowalczyk et al. (2014) reported three newly identified triterpene saponins from the roots of *E. planum* intact plant, namely 3-*O*-*β*-D-glucopyranosyl-(1→2)-*β*-D-glucuronopyranosyl-21-*O*-acetyl-22-*O*-angeloyl-R1-barrigenol (saponin M1), 3-*O*-*β*-D-glucopyranosyl-(1→2)-*β*-D-glucuronopyranosyl-22-*O*-angeloyl-A1-barrigenol (saponin M2B), and 3-*O*-*β*-D-glucopyranosyl-(1→2)-*β*-D-glucuronopyranosyl-22-*O*-angeloyl-R1-barrigenol (saponin M2A) [14]. These saponins were also detected from the in vitro plantlets and dedifferentiated cells of *E. planum*, along with additional saponin M4 (907 *m*/*z*) and saponin M5 (895 *m*/*z*) from the in vitro-derived plants [23]. In this study, the extracts and 80% MeOH fractions of *E. planum* contained saponins, as observed from the TLC plates and UPLC-HRMS/MS chromatograms. The unknown triterpenoid saponins I and II (925 *m*/*z*) were identified, corresponding to the previously reported saponin M1. Similarly, the unknown triterpenoid saponins III and IV (967 *m*/*z*) are likely related to saponin M2A, while unknown triterpenoid saponin V (909 *m*/*z*) matched with saponin M2B (Table 2). Saponins are the largest class of triterpenoids (acid sapogenin) and steroids (natural sapogenin) that are glycosidically connected to a sugar moiety and are classified based on their aglycone structure or sugar moiety [42]. It is worth emphasising that the acylation with angeloyl groups at the C21 or C22 position, together with carbohydrate attachment at the C3, in triterpenoid saponins from *E. planum* is responsible for its cytotoxic efficacy [29].

Triterpenoid saponins were identified as active substances in *L. flos-cuculi.* Early studies reported the presence of gypsogenin, hederagenin, and oleanane-type triterpenoid saponins (coronoside A and coronoside B) in the herb extract of this species [42]. Additionally, Maliński et al. (2021) observed a high concentration of quillaic acid or gypsogenic acid-triterpenoid glycosides in ex vitro roots of *L. flos-cuculi* [25]. Further research revealed that *L. flos-cuculi* cell cultures are also capable of producing triterpenoid saponins [22,28]. Based on the aglycon structure, three classes of saponins were identified from the callus: hydroxygypsogenic acid derivatives, gypsogenic acid derivatives or quillaic acid derivatives, and gypsogenin derivatives. In the present study, triterpenoid saponins were identified from TLC plates and UPLC chromatograms (Figure 1 and Table 2). A high presence of triterpenoid saponins was observed in the cell extract and 80% methanolic fraction. This high abundance of triterpenoid saponins could potentially explain the observed cytotoxic effects.

The MeWo (HTB-65) melanoma cell line, which was used in this study, was derived from the metastatic site and lymph node and exhibited fibroblast morphology (https://www.atcc.org/products/htb-65, accessed on 17 October 2024). The cell line has a *mut*p53E258K mutation, which is responsible for alterations in signalling to cell-cycle arrest, damage repair, senescence, apoptosis, or the modulation of energy metabolism [43]. Moreover, different studies in melanoma have revealed constitutively activated PI3K-AKT-mTOR (AKT) and RAF-MEK-ERK (MAPK) signalling pathways, which contribute to chemoresistance [44]. Saponins have been reported to inhibit various signalling pathways, such as PI3K/AKT, AKT/MAPK, EGFR/PI3K/AKT, PI3K/AKT/mTOR, and RNF6/AKT/mTOR, which leads to its cytotoxic and apoptotic properties on various cancer cell lines [45,46]. The detailed mechanisms of the cytotoxicity of 80% MeOH fractions of *E. planum* and *L. flos-cuculi* cell biomass in MeWo cells needs further investigation.

Numerous studies have identified the presence of phenolic compound content in *E. planum* intact plants, particularly rosmarinic acid, chlorogenic acid, caffeic acid, *p*-coumaric acid, and ferulic acid [15,16,47]. These compounds were found to be retained upon introduction into in vitro conditions (organ and cell cultures) [21,26,27]. Consistent with prior findings, this study also identified rosmarinic acid in EpME, Ep40MF, and Ep80MF (Table 2). Previous molecular studies suggest that rosmarinic acid may be a potential agent for melanoma treatment via the inhibition of cell proliferation and migration and inducing cell apoptosis via gene regulation [48,49]. Similarly, chlorogenic acid was identified in EpME and Ep40MF. This compound potentially suppresses melanogenesis in melanoma cells via tyrosinase inhibition activity, as observed by Li et al. [50].

Our previous studies discovered that the methanolic callus extract of *L. flos-cuculi* shows the presence of *C*-glycoside flavonoids, namely (iso)vitexin derivatives. The identified compounds are mainly apigenin derivatives, annotated as (iso)vitexin *O*-pentoside and (iso)vitexin *O*-rhamnoside, and its acetate and malonylate derivatives. Only one *O*-glycoside flavonoid, rutin, was identified in the methanolic extracts. Other phenolic compounds can be found in derivatives of hydroxycinnamic acids and benzoic acid, 1-*O*-caffeoylglucose, caffeic acid, 4-*O*-glucoside, 1-*O*-dihydroferuloylsucrose, 3-*O*-feruloylsucrose, 2-*O*-feruloyl-malic acid, and hydroxybenzoic acid [28]. Unfortunately, the number and amount of saccharides in the 40% methanolic fractions were enormous and generated high noise during the analysis. Nonetheless, the presence of caffeic acid was identified. Costea et al. (2017) reported that hydroethanolic (50% ethanol) and aqueous freeze-dried extracts from aerial parts of *L. flos-cuculi* showed moderate antioxidant activities in the ABTS+ radical and ferric-reducing power assays. EC_50_ values of 0.1513 ± 0.0123 mg/mL (ABTS+ assay) and 1.6614 ± 0.0246 mg/mL (ferric-reducing power assay) were obtained from aqueous dry extract. Simultaneously, the extraction with 50% hydroalcoholic dry extract exhibited EC_50_ values of 0.2899 ± 0.0184 mg/mL and 1.4832 ± 0.1461 mg/mL for the antioxidant activity assays, respectively [19].

Caffeic acid, which was identified in 40% methanolic fractions of *E. planum* and *L. flos-cuculi*, is known as the most active compound among the hydroxynnamic acids in terms of its cytotoxicity against cancer cells. In this study, caffeic acid, used as a reference compound, demonstrated significant cytotoxicity against MeWo cell lines, reducing the cell viability by up to 60% after 72 h of treatment at a concentration of 50 µg/mL. Previously, it was reported that caffeic acid could induce intracellular GSH depletion by suppressing the reactive oxygen species (ROS) in various human melanoma cells (SK-MEL-5, B16F10, B16-F0, SK-MEL-28, and MeWo) [51,52]. Moreover, Kudugunti et al. observed that caffeic acid enhanced the apoptosis in B16-F0 cells by from five- to seven- fold [52].

Nevertheless, the extracts and fractions from the cell biomass are still rich in several bioactive compounds, which resulted in their working differently than single compounds. The total efficacy of medicinal plant extracts results from the combined action of numerous components which have synergistic, additive, or antagonistic activities [53].

## 5. Conclusions

The present study concludes that cell biomass from *E. planum* and *L. flos-cuculi* could produce valuable secondary metabolites. The methanolic extract derived from the cell biomass of both plants exhibited cytotoxic effects against the MeWo melanoma cell lines, which is the first report from these species. The 40% MeOH fractions showed the presence of phenolic acids and exerted moderate cytotoxicity. However, the results also showed selective characteristics from this fraction against MeWo cell lines compared to the MRC-5 human lung fibroblast cell line. Saponin was detected in the 80% MeOH fractions, which could be the reason for its high toxicity to MeWo cell lines. Further investigation is needed to identify a specific compound or combination of particular compounds in the cell biomass of *E. planum* and *L. flos-cuculi* and understand its mechanism of action. This information will be crucial for effectively utilising both species and plant-cell biotechnology in cancer treatment.

## Figures and Tables

**Table 1:** Retention factors (Rf) from the extracts and fractions of *E. planum* and *L. flos-cuculi* cell biomass in thin-layer chromatogram plates.

Group of Compounds (Rf)	*E. planum*			*L. flos-cuculi*		
	EpME	Ep40MF	Ep80MF	LFcME	LFc40MF	LFc80MF
Phenolic acid	0.10, 0.20, 0.25, 0.83	0.10, 0.20, 0.25, 0.83	0.20, 0.25, 0.33, 0.45, 0.83	0.20, 0.25, 0.83	0.20, 0.25, 0.83	0.25, 0.33, 0.40, 0.48, 0.53, 0.67, 0.83
Saponin	0.42	-	0.22, 0.29, 0.37, 0.44, 0.78	0.32, 0.37	-	0.32, 0.37, 0.44

Legend: methanolic *E. planum* (EpME) and *L. flos-cuculi* (LFcME), 40% methanolic fraction (Ep40MF and LFc40MF), 80% methanolic fraction (Ep80MF and LFc80MF).

**Table 2:** Manually annotated compounds from Ep40MF, Ep80MF, LFc40MF, and LFc80MF. Metabolites were detected using UPLC/HRMS-MS in negative ion mode.

RT	Observed *m*/*z*	Display Formula	Fragmentation	Putative Compound	Ontology	Ref.
	*Ep40MF*					
2.38	115.0023	C_4_H_4_O_4_	71.0123, 115.0023	Maleic acid	Dicarboxylic acids and derivatives	
2.87	117.01797	C_4_H_6_O_4_	73.0279, 116.9272	Methylmalonic acid	Dicarboxylic acids and derivatives	
3.06	191.0191	C_6_H_7_O_7_	85.0279, 87.0072, 111.0073, 191.0190	Citric acid	Carboxylic acid	
3.74	385.13571	C_14_H_26_O_12_	207.0868	Disaccharides	Saccharides	
4.60	587.1836	C_24_H_28_O_10_N_8_	161.0446, 101.0229, 207.0869, 59.0122	Unknown compound I	Other	
4.76	179.0341	C_9_H_8_O_4_	93.0335, 107.0489, 134.0359	trans-Caffeic acid	Hydroxycinnamic acid	
5.67	415.1382	C_22_H_23_O_8_	59.0122, 71.0122, 89.0229, 101.0228, 119.0335, 149.0444, 379.1615	Unknown phenolic compound I	Hydroxycinnamic acid	
5.69	353.0884	C_16_H_17_O_9_	135.0439, 179.0341, 191.0554	Chlorogenic acid	Quinic acids and derivatives	
5.74	167.034	C_8_H_7_O_4_	137.0231, 138.9284, 65.0381, 93.0330, 167.0338, 123.0438	Vanillic acid	M-methoxybenzoic acids and derivatives	
7.23	163.0391	C_9_H_7_O_3_	163.0388, 119.0488, 162.8381	2-Hydroxycinnamic acid	Hydroxycinnamic acids	
7.25	163.0391	C_9_H_8_O_3_	87.5022, 119.0489	trans-Coumaric acid	Hydroxycinnamic acid	
8.16	179.034	C_9_H_7_O_4_	134.9866, 179.0340, 135.0438	Caffeic acid	Hydroxycinnamic acid	
8.24	359.0778	C_18_H_15_O_8_	161.0233, 171.0341, 197.0447	Rosmarinic acid	Hydroxycinnamic acid	
	*Ep80MF*					
5.67	415.1385	C_22_H_23_O_8_	113.5282, 376.9333, 363.7461, 135.7911, 399.6596, 387.2408, 243.9766, 149.0449, 153.0551	Unknown compound II	Other	
6.99	433.1721	C_19_H_29_O_11_	117.0180, 267.0723, 152.9947, 139.1116, 433.1715, 113.0229, 183.1018, 249.0614	Unknown terpenoid I	Terpenoids; Secoiridoid monoterpenoids	
7.39	387.1667	C_18_H_27_O_9_	181.1222, 161.0445, 121.1008, 139.1115, 89.0228, 113.0229, 183.1016, 101.0229	Jasmonic acid glucoside derivative	Other	
7.62	501.1411	C_25_H_25_O_11_	339.0875	Unknown compound III	Phenolics	
7.62	537.1176	C_30_H_15_O_4_N_7_	375.0696, 339.0876	Unknown compound IV	Phenolics	
7.89	515.1564	C_26_H_27_O_11_	173.0447, 179.0341, 191.0553, 338.0800, 353.103	Dicaffeoylquinic acid	Hydrocinnamic acids	
8.19	359.0778	C_18_H_15_O_8_	179.0339, 197.0448, 161.0233	Rosmarinic acid	Hydroxycinnamic acid	
8.45	557.1672	C_28_H_29_O_12_	354.1071, 338.0801, 353.1035	Unknown compound V	Other	
9.79	925.4813	C_40_H_77_O_23_	113.0229, 119.0336, 157.0134, 619.3881, 655.4236, 925.4812	Unknown triterpenoid saponin I	Triterpenoid saponin	[23]
11.23	925.4823	C_40_H_77_O_23_	655.4229, 742.4202, 825.4333, 925.4821	Unknown triterpenoid saponin II	Triterpenoid saponin	[23]
11.52	967.4925	C_49_H_75_O_19_	-	Unknown triterpenoid saponin III	Triterpenoid saponin	[23]
12.08	967.4925	C_49_H_75_O_19_	-	Unknown triterpenoid saponin IV	Triterpenoid saponin	[23]
12.15	909.4871	C_47_H_73_O_17_	-	Unknown triterpenoid saponin V	Triterpenoid saponin	[23]
	*LFc40MF*					
2.31	191.019	C_6_H_7_O_7_	85.0279, 87.0072, 111.0073, 191.0189	Citric acid	Carboxyclic acid	
5.38	303.1454	C_17_H_21_O_4_N	155.0707, 129.0543, 285.1351, 115.0387, 303.1453	Unknown compound VI	Other	
5.47	425.1673	C_17_H_29_O_12_	101.0229,113.0230,161.0444,249.1343,425.1669	alpha-D-Glucopyranosyl 2-O-(2-methylbutanoyl)-alpha-D-glucopyranoside	Other	
5.54	179.0342	C_9_H_7_O_4_	-	Caffeic acid	Hydroxycinnamic acid	
7.47	179.0343	C_9_H_7_O_4_	-	Caffeic acid	Hydroxycinnamic acid	
8.16	187.0969	C_9_H_15_O_4_	123.0801,125.0956,141.0909,187.0211,187.0968	Azelaic acid	other	
8.99	179.0343	C_9_H_7_O_4_	-	Caffeic acid	Hydroxycinnamic acid	
	*LFc80MF*					
8.15	187.0969	C_9_H_15_O_4_	125.0958, 187.0968	Azelaic acid	Carboxyclic acid	
	507.2347	C_25_H_35_O_9_N_2_	115.0387, 143.0335, 338.6758, 338.1740	Unknown compound VII	Amino acid	
	969.4720	C_48_H_73_O_20_	659.3440, 615.3541, 969.4709, 677.3545	Unknown triterpenoid saponin VI	Triterpenoid saponin	
9.15	823.4136	C_42_H_63_O_16_	437.3049, 375.3059, 823.4099, 499.3074, 113.0229, 771.01227, 119.0336, 101.0229, 659.01227	Unknown triterpenoid saponin VII	Triterpenoid saponin	
9.25	601.2050	C_34_H_33_O_10_	269.0569, 225.0666	Unknown compound VIII	Carboxyclic acid	
9.42	516.1629	C_26_H_28_O_11_	261.0674, 304.0731, 336.0994	Unknown compound IX	Phenolic/Flavanoid	
10.13	969.4718	C_66_H_65_O_7_	439.3223, 501.3226	Unknown triterpenoid saponin VIII	Triterpenoid saponin	
10.33	667.2904	C_40_H_43_O_9_	337.114, 113.023, 127.0387, 662.3633, 330.6778, 599.3594, 643.3492, 405.1408, 661.3599, 330.1764	Unknown compound X	Other	
10.64	769.3223	C_44_H_49_O_12_	405.1404, 411.2028, 761.4125	Unknown compound XI	Carboxylic acid	
10.90	688.2957	C_32_H_48_O_16_	599.3599, 643.3497, 405.1409, 661.3599, 330.1765	Unknown compound XII	Other	
11.22	790.3279	C_39_H_52_O_16_N	411.2029	Unknown triterpenoid saponin IX	Triterpenoid saponin	
11.53	709.3011	C_32_H_38_O_18_	330.1765, 661.3600	Isovitexin rhamnoside	Flavonoids	

Legend: methanolic *E. planum* (EpME) and *L. flos-cuculi* (LFcME), 40% methanolic fraction (Ep40MF and LFc40MF), 80% methanolic fraction (Ep80MF and LFc80MF).

**Table 3:** The effect of methanolic extract, including 40% and 80% methanolic fractions, from *E. planum* and *L. flos-cuculi* cell biomass on MRC-5 and MeWo cell lines expressed as IC_50_ values (the concentration at which a substance exerts half its maximal inhibitory effect). Results were obtained from three independent experiments and presented as mean ± SE.

				IC_50_ [µg/mL]				
Cell Lines	Treatment Time	EpME	Ep40MF	Ep80MF	LFcME	LFc40MF	LFc80MF	Caffeic Acid (Reference)
	24 h	178 ± 4	>800	81 ± 9	275 ± 35	>800	68 ± 1	125 ± 5
MRC-5	48 h	135 ± 12	393 ± 32	76 ± 2	186 ± 6	399 ± 3	51 ± 0.5	66 ± 3
	72 h	130 ± 3	267 ± 24	74 ± 4	131 ± 7	266 ± 10	49 ± 1	50 ± 2
	24 h	180 ± 9	484 ± 38	76 ± 2	256 ± 5	271 ± 7	65 ± 0.5	135 ± 8
MeWo	48 h	121 ± 3	210 ± 8	71 ± 3	178 ± 13	233 ± 13	63 ± 5	41 ± 3
	72 h	103 ± 4	145 ± 10	52 ± 4	147 ± 4	172 ± 7	47 ± 0.5	25.4 ± 2

## Data Availability

Data are contained within the article.
